# Plasmon-Modulated Excitation-Dependent Fluorescence from Activated CTAB Molecules Strongly Coupled to Gold Nanoparticles

**DOI:** 10.1038/srep43282

**Published:** 2017-03-07

**Authors:** Si-Jing Ding, Fan Nan, Xiao-Li Liu, Zhong-Hua Hao, Li Zhou, Jie Zeng, Hong-Xing Xu, Wei Zhang, Qu-Quan Wang

**Affiliations:** 1 Department of Physics, Key Laboratory of Artificial Micro- and Nano-Structures of Ministry of Education, Wuhan University, Wuhan 430072, P. R. China; 2 Hefei National Laboratory for Physical Sciences at the Microscale, Department of Chemical Physics, University of Science and Technology of China, Hefei, Anhui 230026, P. R. China; 3 Institute of Applied Physics and Computational Mathematics, Beijing 100088, P. R. China; 4 The Institute for Advanced Studies, Wuhan University, Wuhan 430072, P. R. China

## Abstract

Excitation-dependent fluorophores (EDFs) have been attracted increasing attention owing to their high tunability of emissions and prospective applications ranging from multicolor patterning to bio-imaging. Here, we report tunable fluorescence with quenching dip induced by strong coupling of exciton and plasmon in the hybrid nanostructure of CTAB* EDFs and gold nanoparticles (AuNPs). The quenching dip in the fluorescence spectrum is tuned by adjusting excitation wavelength as well as plasmon resonance and concentration of AuNPs. The observed excitation-dependent emission spectra with quenching dip are theoretically reproduced and revealed to be induced by resonant energy transfer from multilevel EDFs with wider width channels to plasmonic AuNPs. These findings provide a new approach to prepare EDF molecules and a strategy to modulate fluorescence spectrum via exciton-to-plasmon energy transfer.

The excitation-dependent fluorophores (EDFs) have received considerable attention because of their tunable fluorescence spectrum with some unique advantages and prospective applications in material sciences and biology^1^,^2^,^3^,^4^,^5^,^6^,^7^,^8^,^9^. The emission peak of EDFs red-shifts as the excitation wavelength increases, which is usually explained as the red-edge effect in the multilevels^10^,^11^,^12^,^13^. Several remarkable examples of EDFs reported in the literature include fluorescent polar molecules and carbon-based nanomaterials, such as carbon nanotubes^14^,^15^,^16^, carbon quantum dots^7^,^17^,^18^,^19^,^20^,^21^,^22^,^23^,^24^, graphene quantum dots (G-QDs)^25^,^26^,^27^,^28^,^29^,^30^, and pyromellitic diimide nanowires^31^. Very recently, hydrogen-bonded monoacylglycerol clusters as a new kind of EDFs is also reported^32^.

The emission spectrum of EDFs can not only be tuned by the excitation field, but also be manipulated by coupling of surface plasmon of metal nanostructures, which is widely used to diversify the types of light-matter interactions at nanometer scale^33^,^34^,^35^,^36^,^37^,^38^,^39^,^40^,^41^,^42^. The fluorescence efficiency of nanoemitters can be enhanced or quenched by metal nanoparticles depending on factors, such as the identity of the metal, the distance of the fluorophore to the nanoparticles, and the orientation of its transition dipole^43^,^44^. The fluorescence quenching effect induced by energy and electron transfer to metallic species has been reported in the literature^45^,^46^,^47^,^48^,^49^,^50^, which has applications ranging from plasmon loss compensation to bio-sensor. It has been revealed that the quenching efficiency depends on the spectral overlap and particle sizes^51^. More intriguingly, the quenching distance depends strongly on the intrinsic nonradiative relaxation and level structures of the fluorophores. The typical fluorescence quenching distance between organic molecules and metal nanoparticles reaches ~10 nm^52^. A few remarkable works on plasmon-enhanced fluorescence of EDFs have been reported by using carbon quantum dots and nanotubes^14^,^53^,^54^. For instance, the surfactant coated carbon nanotubes directly contacted with gold film exhibit strong enhanced fluorescence without quenching. However, the energy transfer mechanism of strongly coupled EDFs and plasmonic nanostructures is unclear^14^.

Herein, we report plasmon-modulated excitation-dependent fluorescence from the nanocomplex CTAB*@AuNPs of activated hexadecyltrimethyl ammonium bromide (CTAB) ligands strongly coupled to Au nanoparticles. The excitation-dependent fluorescence of CTAB* EDF molecules is revealed to be assigned to the generated –C=O group during hydrothermal process. The shape and plasmon resonance of AuNPs is also finely tuned by hydrothermal reaction in the presence of CTAB molecules. A fluorescence quenching dip is observed in the strongly coupled CTAB*@AuNPs. To reveal the mechanism and consequence of the resonant energy transfer from CTAB* EDFs to AuNPs, we propose a theoretical model consisting multilevel intermediate states for the excitation-dependent fluorescence of CTAB*, which is coupled to plasmon resonance of AuNPs and the calculated fluorescence spectrum with Fano profile coincided with the experimental results very well.

## Results

### Absorption and excitation-dependent fluorescence of CTAB* molecules

**Figure 1:**
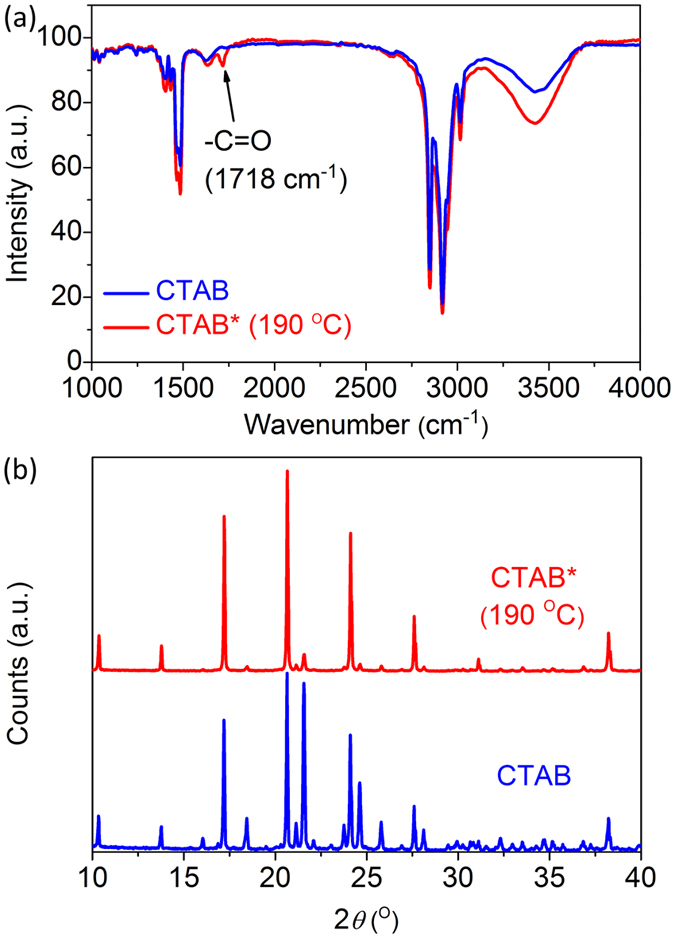
FITR spectrum (**a**) and XRD pattern (**b**) of CTAB and CTAB* molecules treated at *T*_react_ = 190 °C. A new resonance mode appears at 1718 cm^−1^ in FITR of CTAB*, which is assigned to the generated –C=O during hydrothermal process. In XRD pattern, the side peaks are significantly suppressed and no new peaks are generated after hydrothermal reaction.

The EDFs used in this study, marked by CTAB*, is prepared by hydrothermal reaction of CTAB ligand molecules. CTAB is a popular ionic surfactant used for either stabilizing or reshaping AuNPs during synthesis and post-thermal processes^55^,^56^,^57^,^58^,^59^,^60^; CTAB* has prominent advantages of chromophore generation as well as strong exciton-plasmon coupling with AuNPs. In the Fourier transform infrared (FTIR) spectrum as shown in Fig. 1a, all absorption bands of CTAB molecules remain after the hydrothermal reaction, but a new absorption band at 1718 cm^−1^ appears, the corresponding strength of which prominently increases with reaction temperature *T*_react_ (also see Supplementary Information), indicating the generation of –C=O group in CTAB* molecules. In the X-ray diffraction (XRD) patterns (Fig. 1b), the periodical main peaks at 2*θ* = 10.35, 13.77, 17.21, 20.66, 24.12, 27.60, and 31.11° of CTAB molecules remains unchanged after hydrothermal reaction, but the other side peaks are significantly suppressed and no new peaks are generated, which indicates that CTAB and CTAB* molecules have same crystal phase.

**Figure 2:**
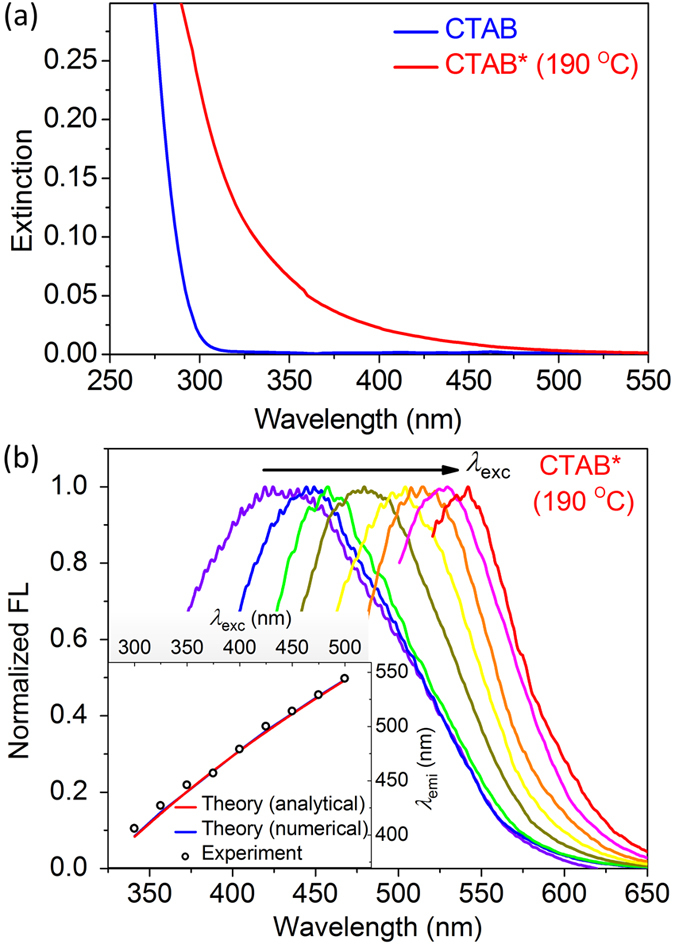
Absorption and excitation-dependent fluorescence spectra of CTAB* molecules at *T*_react_ = 190 °C. (**a**) Absorption spectra of the CTAB and CTAB* molecules. (**b**) Normalized excitation-dependent fluorescence spectra of the CTAB* EDFs with *λ*_exc_ = 325, 350, 375, 400, 425, 450, 475, and 500 nm. Inset: Relationship of excitation and emission wavelength of CTAB* molecules.

CTAB* molecules have much stronger absorption in the violet region than that of unactivated CTAB molecules. The absorption band red-shifts to 400 nm, while the absorption tail also prominently increases and extends to 550 nm as the reaction temperature *T*_react_ increases to 190 °C (Fig. 2a). Interestingly, the hydrothermally reacted CTAB* molecules exhibit strong fluorescence with a broad emission spectrum almost covering the whole visible region. The fluorescence intensity increases approximately one order of magnitude as *T*_react_ increases from 130 °C to 190 °C. The excitation-dependent fluorescence of CTAB* molecules is assigned to the surface states related to –C=O group generated by hydrothermal reaction. Moreover, the emission peak wavelength (*λ*_emi_) red-shifts from 405 nm to 545 nm as the excitation wavelength (*λ*_exc_) increases from 300 nm to 500 nm (Fig. 2b), while the corresponding fluorescence Stokes shift Δ decreases from 1150 meV to 160 meV. It is worth noting that the *λ*_exc_ ~ *λ*_emi_ dependence of CTAB* molecules is surprisingly similar to that of G-QDs reported in the literature^28^, which suggests the similar origin of excitation-dependent fluorescence of CTAB* with that of G-QDs.

### Hydrothermally reshaping Au nanorods and preparation of CTAB*@AuNPs nanocomplex

**Figure 3:**
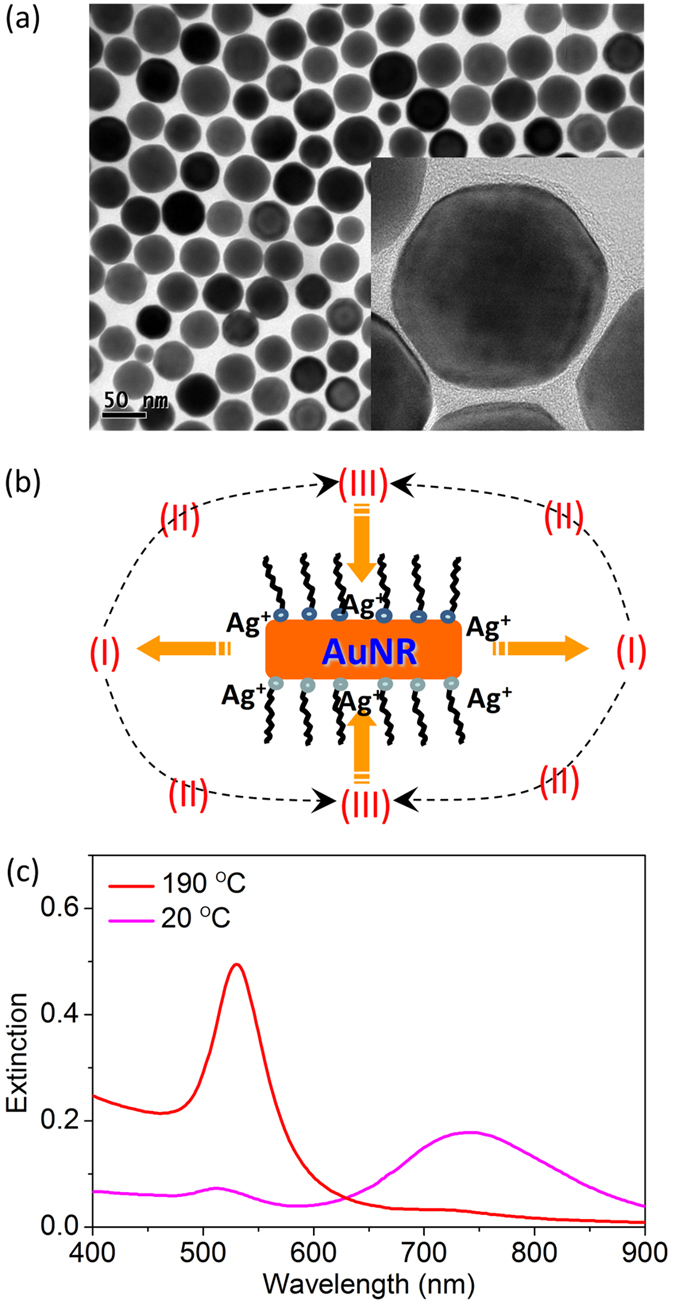
TEM and extinction spectrum of CTAB*@AuNPs annealed at *T*_react_ = 190 °C. (**a**) TEM image of spherical AuNPs. Inset showes a very thin layer of ligand molecules covered on the surface of AuNPs. (**b**) Illustration of three chemical processes of reshaping gold nanorods to nanospheres. I: Au^0^ + 4Br^−^ → AuBr_4_^−^ + 3e^−^; II: AuBr_4_^−^ + CTA^+^→ [CTA^+^] [AuBr_4_^−^]; III: [CTA^+^] [AuBr_4_^−^] + 3e^−^ → Au^(0)^ + [CTA^+^] + 4Br^−^. (**c**) Extinction spectra of initial gold nanorods and spherical AuNPs.

The nanocomplexes of CTAB*@AuNPs are also prepared by hydrothermal reaction at the same condition. CTAB molecules are popular stabilizer in synthesis of gold nanorods and act as soft templates to confine the gold growth along one dimension^55^,^56^,^57^, which also reshape and etch nanorods during the post-thermal annealing process^58^,^59^,^60^. The initial gold nanorods synthesized at the room temperature have average length (*L*_Au_) of ~47 nm and average diameter (*d*_Au_) of ~14 nm. The transformation from nanorods to spherical nanoparticles is observed when the reaction temperature *T*_react_ increases to 190 °C. Figure 3a presents the transmission electron microscope (TEM) image of the annealed AuNPs (*T*_react_ = 190 °C) with a average diameter of ~50 nm and a very thin layer of ligand molecules (see the inset).

The chemical processes of transformation from nanorods to spherical nanoparticles are illustrated in Fig. 3b. As the reaction temperature *T*_react_ increases, the length of Au nanorods (AuNRs) is decreased by the etching effect of CTAB on the nanorod ends (process I: Au^0^ + 4Br^−^ → AuBr_4_^−^ + 3e^−^), while the diameter prominently is increased by the re-deposition of Au on the nanorod sides (process III: [CTA^+^] [AuBr_4_^−^] + 3e^−^ → Au^(0)^ + [CTA^+^] + 4Br^−^). Such reshaping processes of AuNRs are very similar to those of Au nanostars, which can be explained by Ostwald ripening involving dissolution of weakly bound surface atoms at areas with high convex curvature and re-deposition at areas with low convex curvature^58^. The thermally induced desorption and oxidation of CTAB ligand on the side facets enhances gold growth on the sides of AuNRs^60^.

The longitudinal and transverse surface plasmon resonance (L-SPR and T-SPR) wavelengths of the initial gold nanorods are located at 712 nm and 518 nm, respectively. L-SPR wavelength blue-shifts and T-SPR strength prominently increases as *T*_react_ increases. When *T*_react_ increases to 190 °C, the spherical AuNPs exhibits a single SPR mode at the wavelength of ~530 nm (Fig. 3c).

### Tunable fluorescence spectrum of CTAB*@AuNPs suspensions

**Figure 4:**
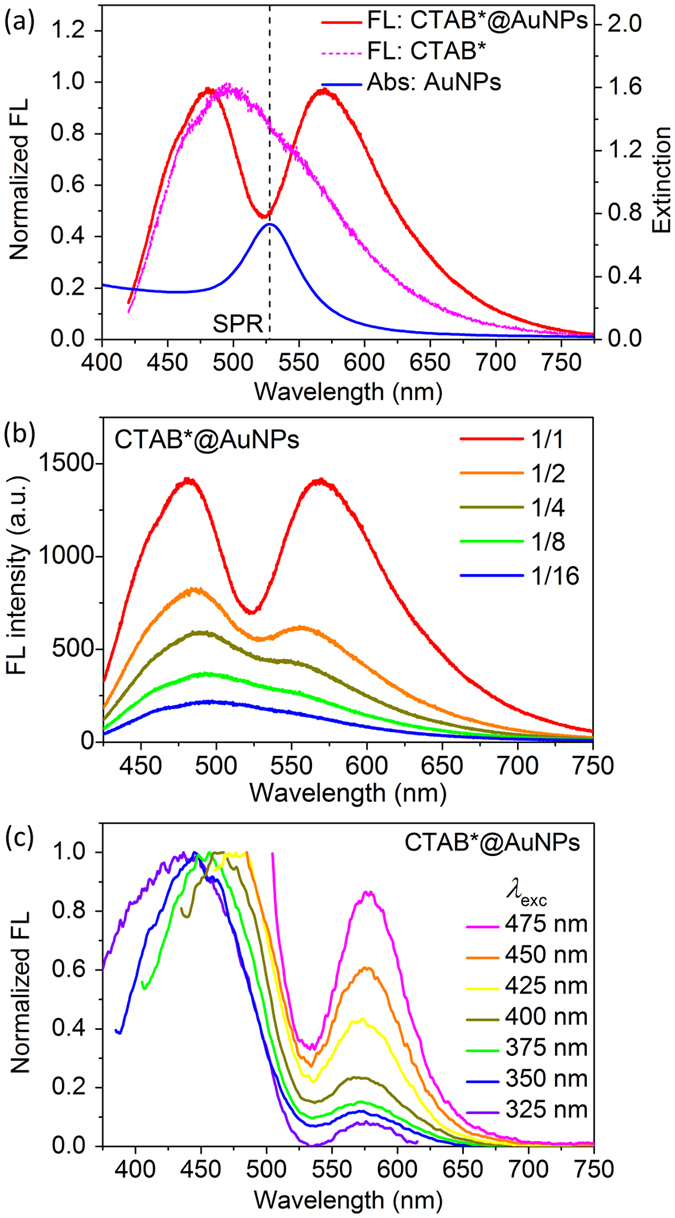
Plasmon-modulated fluorescence spectrum of CTAB*@AuNPs. (**a**) Normalized fluorescence spectra of pure CTAB* molecules and CTAB*@AuNPs annealed at *T*_react_ = 190 °C. (**b**) Concentration-dependent fluorescence spectrum of CTAB*@AuNPs. The dilution ratios of five samples are 1/1, 1/2, 1/4, 1/8, and 1/16. The dip depth and right peak intensity nonlinearly increasing with the concentration indicates strong coupling and energy transfer of CTAB*@AuNPs. (**c**) Excitation-dependent fluorescence spectrum of CTAB*@AuNPs with *λ*_exc_ = 325, 350, 375, 400, 425, 450, and 475 nm. A dip appears around the LSPR of AuNPs and the relative intensity of right peak increases with *λ*_exc_.

Eventually, the nanocomplexes of CTAB*@AuNPs with strong exciton-plasmon coupling are obtained, which exhibit plasmon-modulated fluorescence spectrum. Figure 4a demonstrates a dip in the fluorescence spectrum at the SPR of AuNPs (~530 nm). This prominent dip could not be simply explained by the reabsorption of the AuNPs. Figure 4b presents concentration-dependent fluorescence spectrum of CTAB*@AuNPs, it shows that the dip depth and the low-energy fluorescence peak intensity nonlinearly increasing with the concentration.

Figure 4c presents the fluorescence spectra of CTAB*@AuNPs with different excitation wavelengths. The spectra acquired with excitation wavelength *λ*_exc_ = 325~425 nm are normalized by the emission intensity of high-energy peak. The fluorescence intensity at the dip is approximately linearly dependent on the excitation wavelength, and this linear dependence is used to estimate the relative intensity of low-energy peak when *λ*_exc_ = 450 and 475 nm (where the excitation wavelength is very close to the position of high-energy peak). Figure 4c clearly shows that the position of high-energy peak red-shifts as the excitation wavelength increases, but the positions of dip and low-energy peak keep almost unchanged. The fluorescein isothiocyanate (FITC) molecules also have a fluorescence peak at around 520 nm, but it is independent on the excitation wavelength, the quenching dip is not observed in the fluorescence spectrum of the FITC molecules strongly coupled to AuNPs^52^.

There are three possible physical mechanisms to induce a dip (with two peaks) in fluorescence spectrum. (i) Radiation re-absorption effect. The radiative emissions are absorbed by plasmon resonance of AuNPs, this radiative energy transfer process will be approximately linearly dependent on the concentration of AuNPs. (ii) Rabi splitting of two strongly coupled radiative dipoles. In this case, two splitting peaks are induced by ultrafast energy exchanges between exciton and plasmon, the position of low-energy peak will prominently red-shift as the excitation wavelength increases. (iii) Nonradiative energy transfer from CTAB* to AuNPs. The efficiency of this resonant energy transfer dramatically increases with the concentration of AuNPs, and the positions of dip and low-energy peak is independent on the excitation wavelength. These two behaviors well coincide with the experimental results presented in Fig. 4b,c. Therefore, we conclude that the fluorescence dip of CTAB*@AuNPs is attributed to strong coupling of exciton-plasmon and efficient nonradiative energy transfer.

### Theoretical analysis of energy transfer from EDF to AuNP

Then, we propose a theoretical model to describe multilevel EDFs and calculate excitation-dependent fluorescence spectra of CTAB* EDF with and without AuNP. The basic physical picture for pure EDFs (without AuNP) is the presence of long lifetime multilevel states due to the surface states from functional group^7^. The laser field with frequency *ω*_*exc*_ excites the state |*ω*_*exc*_〉 of EDFs, and then the electrons in the state |*ω*_*exc*_〉 relax to the lower multilevel intermediate states |*ħϖ*〉. The long-lived electrons on the intermediate states emit photons with frequency between *ω*_*low*_ and *ω*_*exc*_, here *ω*_*low*_ is the frequency of photons emitted from the lowest state with frequency *ω*_*low*_ relative to the HOMO of EDFs. Then the total fluorescence spectrum can be calculated as


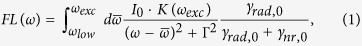


where *I*_0_ is the intensity of the incident excitation field, *FL*(*ω*) is the fluorescence intensity at the emission frequency *ω*, Γ the energy level broadening of the intermediate states. *K*(*ω*_*exc*_) = 1/[(*ω*_*exc*_ − *ω*_0_)^2^ + *η*^2^] describes the excitation efficiency, and the state with energy *ħω*_0_ (=3.312 eV) has the highest excitation efficiency according to the experimental observation, *γ*_*rad*,0_ and *γ*_*nr*,0_ represents the radiative and nonradiative rate, respectively. For simplicity, we have assumed the same radiative/nonradiative rate and also the same spectral width for all intermediate states. When Γ is small, we find that the fluorescence peak appears at the wavelength,


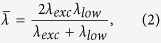


Equation (2) clearly demonstrates that the fluorescence peak wavelength increases with the excitation wavelength. The calculated emission peaks based on this analytical formula agrees well with the experimental results (see inset of Fig. 2b).

In the presence of AuNPs, the surface plasmon resonance leads to the local field enhancement *I* = *I*_0_ · *P*(*ω*_*exc*_), while the radiative rate of EDFs changes to *γ*_*rad*_ = *γ*_*rad*,0_ · *P*(*ω*). The local field enhancement factor, *P*(*ω*), is defined as *P*(*ω*) = 1 + *q* · |*β*(*ω*)|^2^, *β* = (*ε* − *ε*_*m*_)/(*ε* + 2*ε*_*m*_), where *ε* and *ε*_*m*_ are the dielectric constants of gold and the medium, respectively. Moreover, the interaction between AuNPs and EDFs leads to an additional energy transfer channel with rate *γ*_*M*_ ∝ Im(*β*)^61^. In total, the emission spectrum of the EDF@AuNPs is


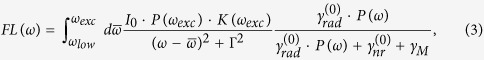


**Figure 5:**
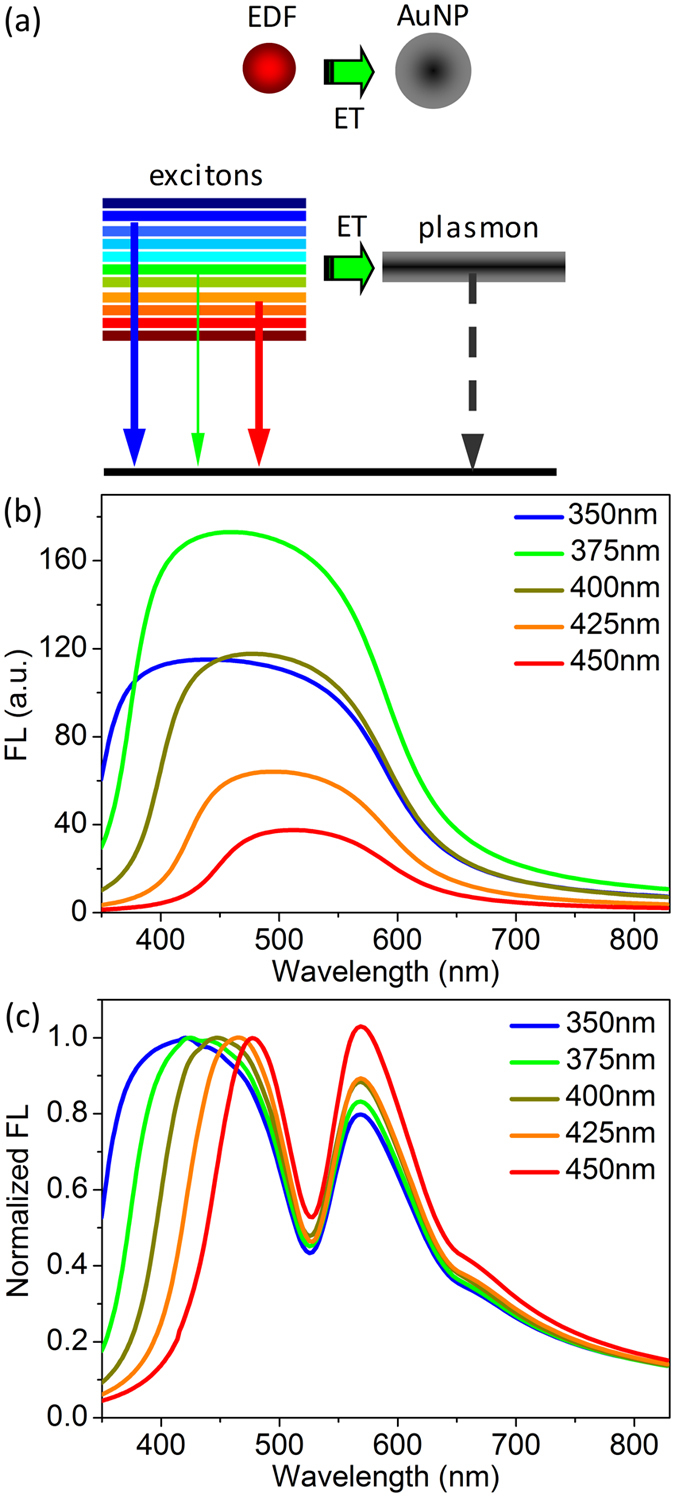
Theoretical model and calculated fluorescence spectrum of EDF@AuNP. (**a**) Illustration of multi-level radiative emissions and energy transfer of the EDF@AuNP nanocomplex. (**b**) Calculated fluorescence spectrum of the EDFs with different excitation wavelength. (**c**) Plasmon-modulated excitation-dependent fluorescence spectrum of EDF@AuNP, which is normalized by the left peak intensity.

Figure 5a illustrates the model of energy transfer from an EDF with multilevels to an AuNP with a plasmon resonance mode. Figure 5b gives the calculated fluorescence spectrum of a pure EDF with excitation wavelength of 350, 375, 400, 425, and 450 nm, which qualitatively describes the major behaviors of excitation-dependent fluorescence of CTAB* molecules.

The quenching dip at the SPR is well reproduced in the calculated fluorescence spectrum of an EDF strongly coupled with an AuNP. Figure 5c demonstrates that the relative peak intensity with low-energy increasing with excitation wavelength. These theoretically calculated emission spectra qualitatively agree with the experimental observations.

### Comparing energy transfer dip in absorption and fluorescence spectrum

It is interesting to note that the existence of Fano profile is due to the interaction between the molecular channel with wide width and the plasmon channel with narrower width in the absorption spectrum. It is different from many cases studied before^62^,^63^, where plasmons and excitons in semiconductor quantum dots/molecules play the opposite roles, i.e., the plasmon provides the wide width channel and the semiconductor quantum dots/molecules provide the narrow width channel. The Fano dip in the absorption spectrum of plamon-molecule hybrids is located at the narrow absorption band of molecules and induced by the energy transfer from plasmons to molecules (i.e. plasmon → exciton process)^62^,^63^,^64^,^65^,^66^,^67^. In this case, the fluorescence dip located at the SPR wavelength is induced by energy transfer from fluorescent EDFs to plasmonic AuNPs (i.e. exciton → plasmon process). However, both dips in absorption and emission spectrum are induced by energy transfer but with the opposite direction of energy flowing.

## Conclusions

In summary, a new kind of EDFs, CTAB* molecules, are prepared by hydrothermal reaction of CTAB molecules, exhibiting similar dependence of excitation-emission wavelength comparing to G-QDs. The origin of the CTAB* fluorescence is revealed, which is assigned to the newly generated surface states caused by –C=O group. The nanocomplexes of CTAB*@AuNPs with strong exciton-plasmon coupling are also prepared by the hydrothermal process, the shape as well as plasmon resonance of which is precisely tuned by adjusting hydrothermal reaction temperature. A prominent quenching dip is observed in the fluorescence spectrum of CTAB*@AuNPs, which is highly tunable by adjusting concentration and excitation wavelength and power. This unique excitation-dependent emission spectrum with Fano profile is well reproduced by theoretical calculations, and is revealed to be induced by efficient resonant energy transfer from multilevel CTAB* molecules to AuNPs. These results provide a new approach to prepare EDFs with –C=O chromophores and a strategy to modulate fluorescence spectrum in strong coupling regime via exciton → plasmon and exciton → plasmon → exciton energy transfer.

## Methods

### Sample Preparation

Chloroauric acid (HAuCl_4_ · 4H_2_O, 99.99%), silver nitrate (AgNO_3_, 99.8%), hydrochloric acid (36~38%), and sodium borohydride (NaBH_4_, 96%) were all purchased from Sinopharm Chemical Reagent Co. Ltd. (Shanghai, China). CTAB (99.0%) was obtained from Amresco, Inc. (America). All chemicals were used as received and without further purification. The water used in all reactions was obtained by filtering through a set of millipore cartridges (Epure, Dubuque, IA).

The Au nanorods were prepared using a seed-mediated growth method^56^. The Au seed solution was made by adding 600 μL of ice-cooled NaBH_4_ solution (10 mM) into a 10 mL aqueous solution containing HAuCl_4_ (5 mM) and CTAB (200 mM). For the synthesis of Au nanorods, 1.2 mL of aqueous HAuCl_4_ solution, 8 μL of aqueous AgNO_3_ solution, 7 μL of aqueous HCl solution, and 0.66 mL of aqueous ascorbic acid solution were mixed, followed by the addition of 8 μL of the Au seed solution. The concentration of Au nanorods was estimated to be about 8.0 nM according to measured extinction coefficients at the localized surface plasmon resonance peak wavelength^57^. Subsequently, the above Au nanorods was poured into a stainless steel autoclave and reshaped to Au nanospheres via annealing processes. Finally, the reactor was automatically cooled to room temperature. The resulting solution was centrifuged at 16,000 rpm to separate supernate and deposit for further characterization.

### Sample Characterization

The TEM and scanning electron microscopy (SEM) images were measured with a JEOL 2010 HT and a Hitachi S-4800. The absorption spectra were taken on a TU-1810 UV-Vis-NIR spectrophotometer (Purkinje General Instrument Co. Ltd. Beijing, China). The fluorescence signal of the samples is excited by a pulsed laser, collected in reflection geometry, and recorded by a spectrometer (Spectrapro 2500i, Acton) with a liquid-nitrogen-cooled charge-coupled device (SPEC-10, Princeton). The pulsed laser is provided by a mode-locked Ti:Sapphire laser (Mira 900, Coherent) equipped with an optical frequency doubling system.

## Additional Information

**How to cite this article:** Ding, S.-J. *et al*. Plasmon-Modulated Excitation-Dependent Fluorescence from Activated CTAB Molecules Strongly Coupled to Gold Nanoparticles. *Sci. Rep.* **7**, 43282; doi: 10.1038/srep43282 (2017).

**Publisher's note:** Springer Nature remains neutral with regard to jurisdictional claims in published maps and institutional affiliations.

## Supplementary Material

Supplementary Information
