# Supplementary material for: Plasmon-Modulated Excitation-Dependent Fluorescence from Activated CTAB Molecules Strongly Coupled to Gold Nanoparticles
Source: Sci Rep. 2017 Mar 7;7:43282. doi: 10.1038/srep43282 (PMC5339731; doi:10.1038/srep43282)
Supplement: Supplementary Information [file srep43282-s1.doc]

Supplementary Information for

**Plasmon-Modulated Excitation-Dependent Fluorescence from Activated CTAB Molecules Strongly Coupled to Gold Nanoparticles**

*Si-Jing Ding,^a^*^‡^ *Fan Nan,^a^*^‡^ *Xiao-Li Liu,^a^ Zhong-Hua Hao,^a^ Li Zhou,^a^ Jie Zeng,^b^ Hong-Xing Xu,^a,d^ Wei Zhang***^c^ and Qu-Quan Wang***^a,d^*

* Email: [qqwang@whu.edu.cn](mailto:qqwang@whu.edu.cn), zhang_wei@iapcm.ac.cn


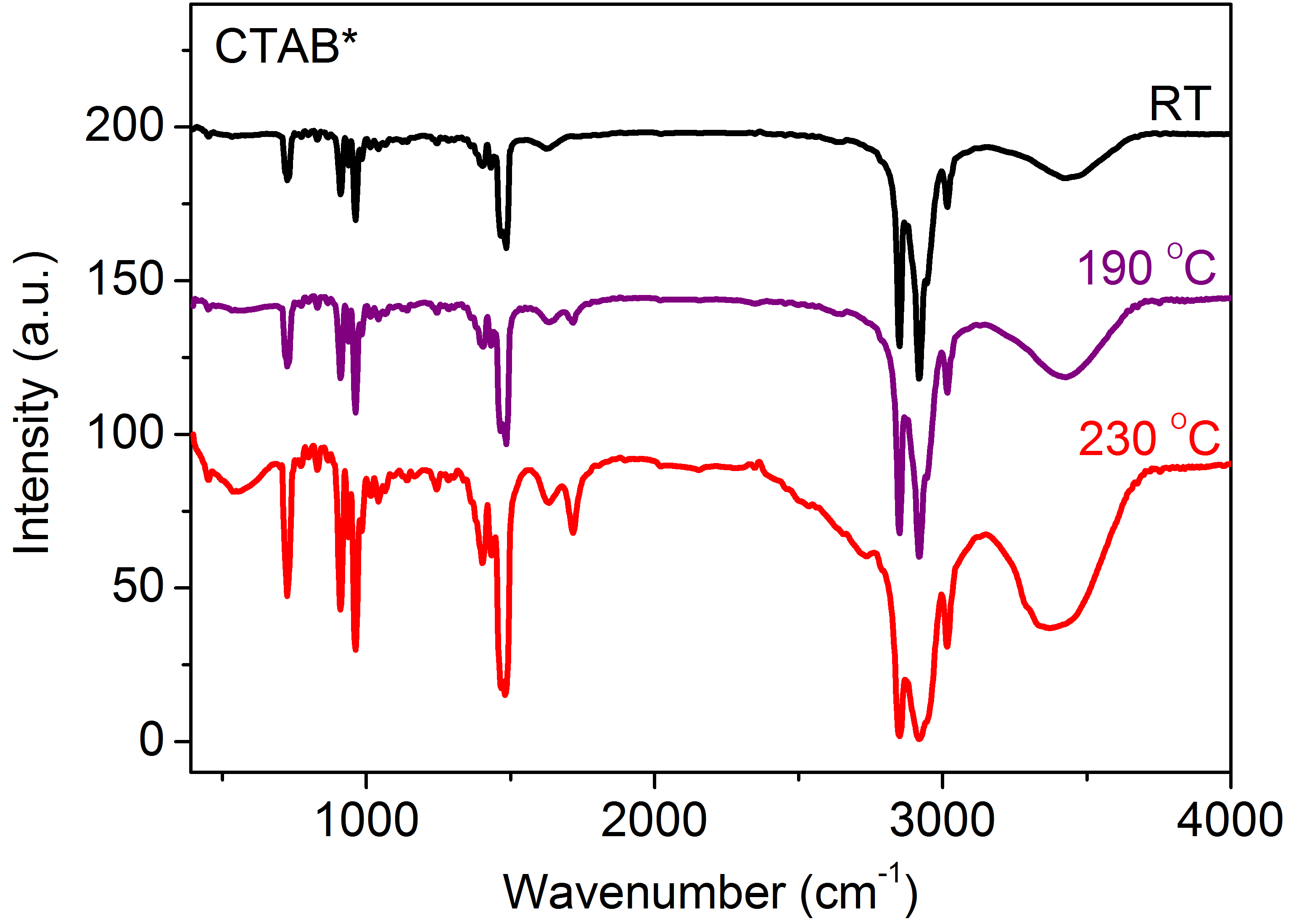


**Figure S1**. FITR spectra of CTAB and CTAB* at the reaction temperature *T*_react_ = 190 and 230 °C.


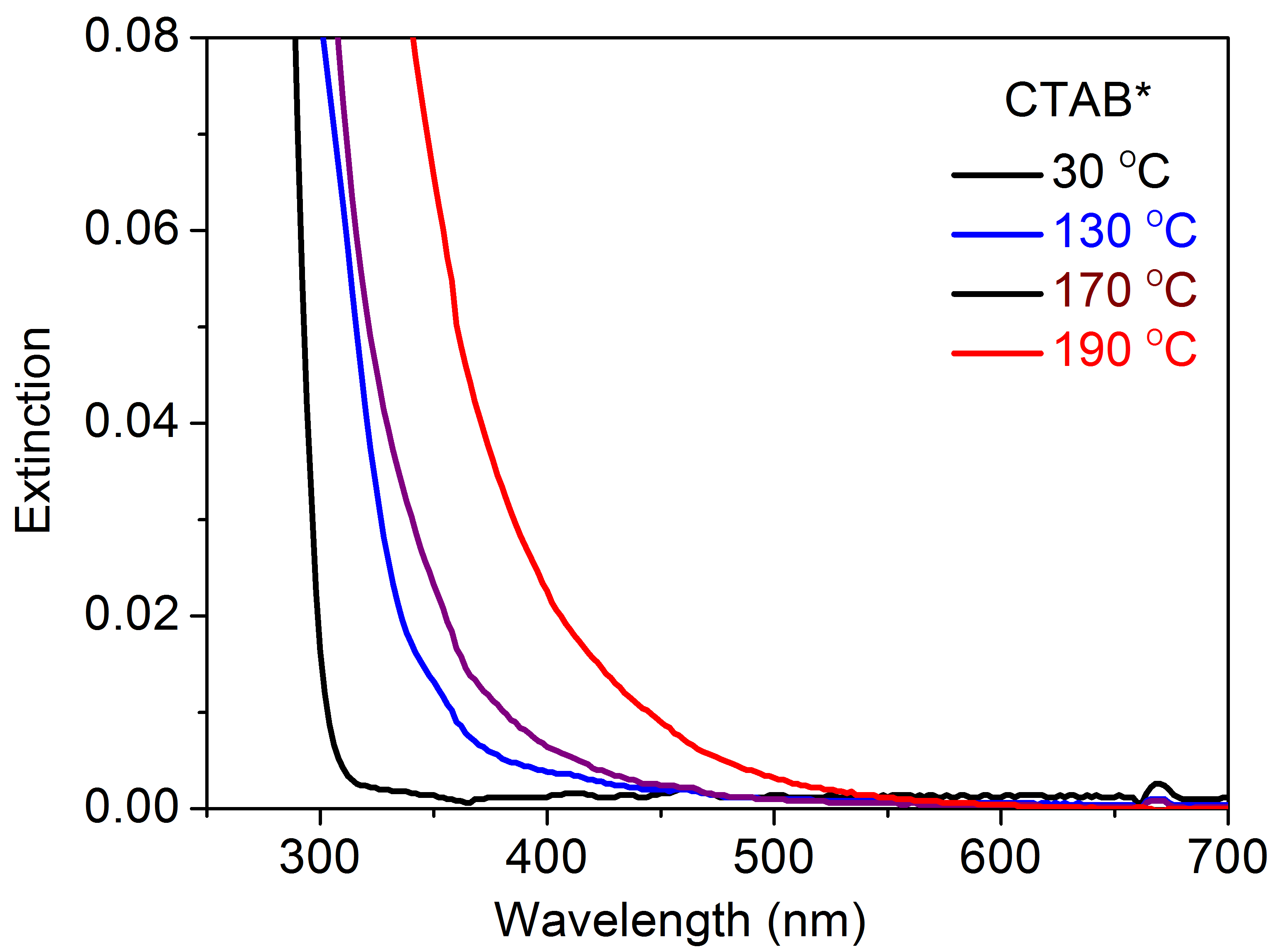


**Figure S2**. Absorption spectra of CTAB and CTAB* molecules at the reaction temperature *T*_react_ = 130, 170, and 190 °C.


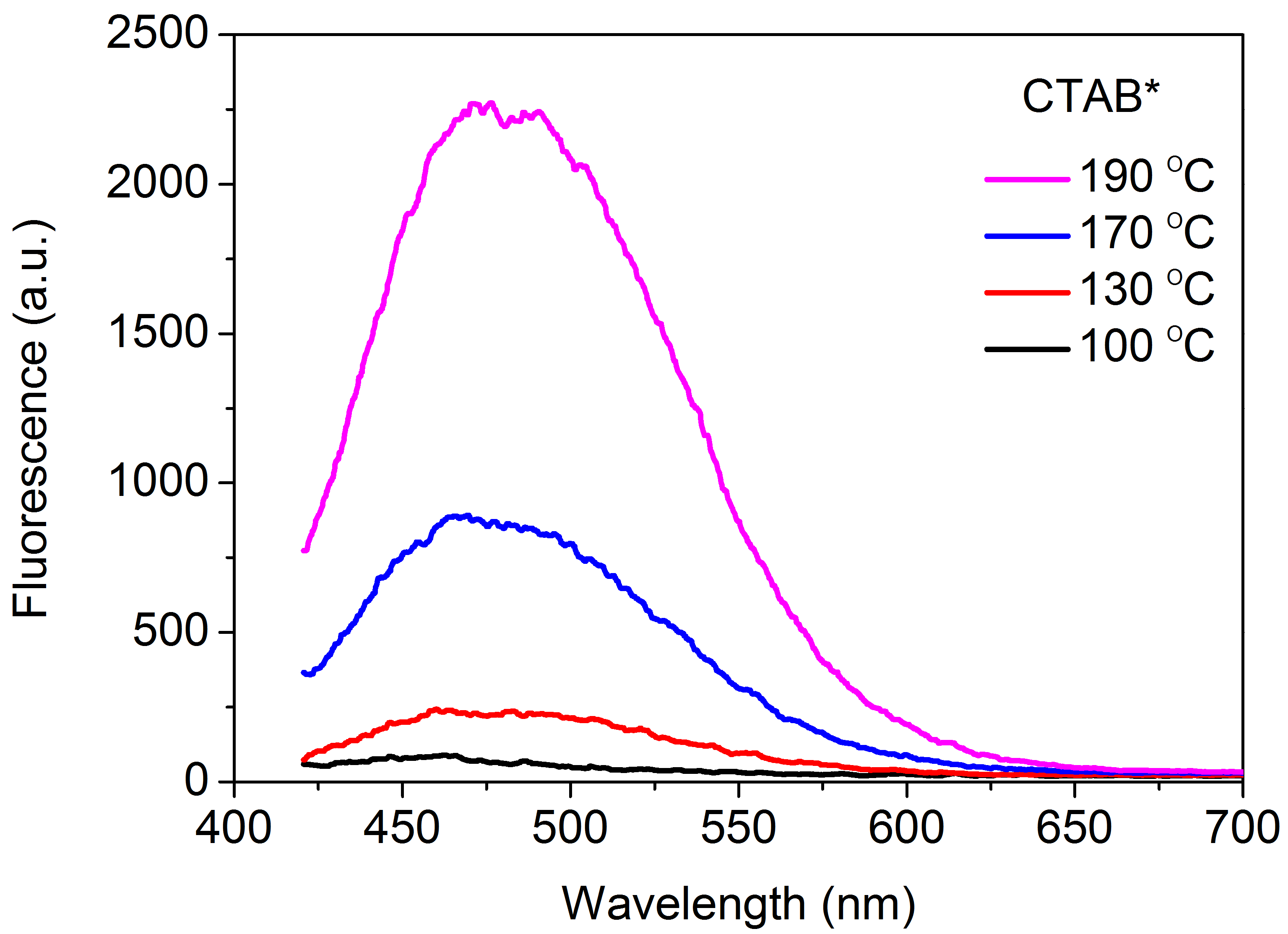


**Figure S3**. Fluorescence spectra of CTAB* molecules at the reaction temperature *T*_react_ = 100, 130, 170, and 190 °C.


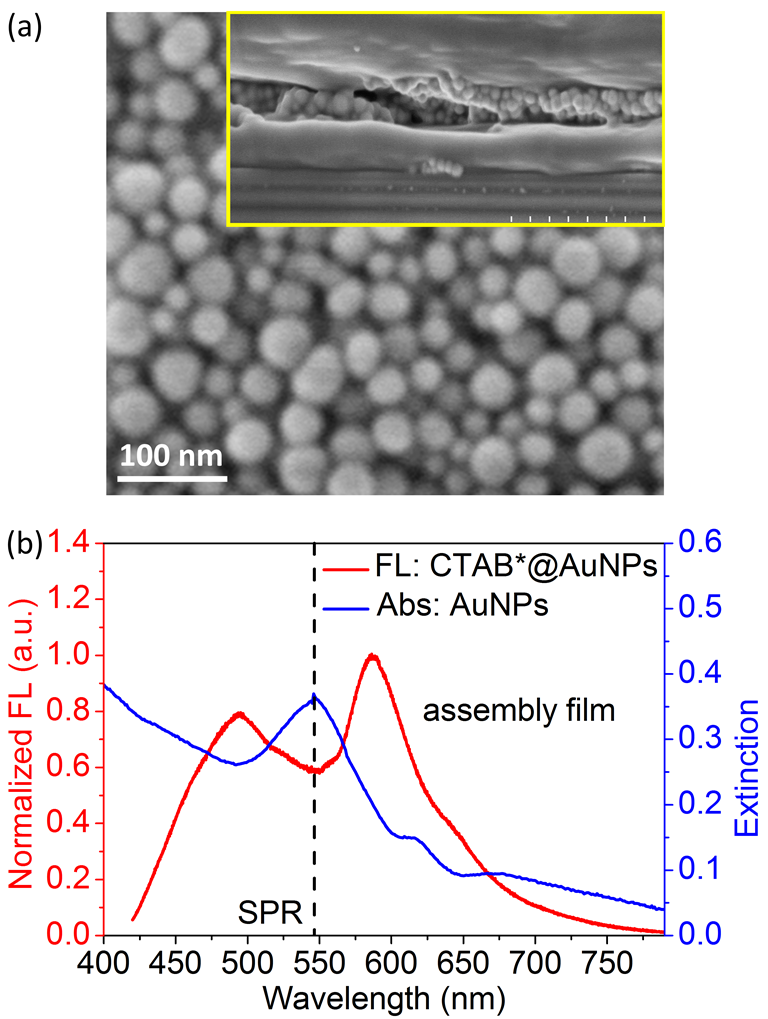


**Figure S4.** Plasmon-modulated fluorescence of CTAB*@AuNPs assembly film. (a) SEM image of CTAB*@AuNPs assembly film. (b) Absorption and fluorescence spectra of the CTAB*@AuNPs assembly film. Both absorption peak and the emission dip red-shift to ~545 nm.
